# Empathy toward others’ distress and its determinants among psychiatry trainees in Tunisia

**DOI:** 10.1192/j.eurpsy.2025.1645

**Published:** 2025-08-26

**Authors:** Y. Trabelsi, Z. Bouzaabia, B. N. Saguem, N. Jaafar

**Affiliations:** 1 Psychiatry Department, Farhat Hached Hospital, sousse, Tunisia

## Abstract

**Introduction:**

Empathy, a key component of an effective physician-patient relationship, is understood as a multifaceted socio-cognitive ability influenced by various cognitive, emotional, social factors. While it has been extensively studied in medical students and healthcare professionals overall, there is limited research on empathy skills among psychiatrists, whose profession especially demands a strong capacity for empathy in their practice.

**Objectives:**

This study aimed to explore the different facets of empathy among Tunisian psychiatry trainees and assess their connections with demographic, occupational, emotional, and social variables.

**Methods:**

Across-sectional study was conducted. An online survey was proposed to 120 Tunisian psychiatry trainees. In addition to sociodemographic and work-related variables, it comprised the Davis’s Interpersonal Reactivity Index, a 28-item tool used for a multidimensional assessment of empathy with four distinct subscales: Perspective taking(PT), Empathic concern(EC), Personal distress(PD) and Fantasy scale(FS). The survey also included the Difficulties in Emotion Regulation Scale(DERS), the Perceived Stress Scale(PSS), the Social Support Questionnaire(SSQ) and the General Self-Efficacy Scale(SE). Factors associated with empathy were evaluated using t test/ANOVA for categorical variables and correlation for continuous predictors.

**Results:**

The total participation rate was 71% and the mean empathy subscores were as follows: 19.04 ± 3.95 for PT, 20.41 ± 3.71 for EC, 12.67 ± 4.41 for PD and 16.40 ± 4.91 for FS. Empathy dimensions’ scores were significantly associated with the rank in the siblings, mother’s level of education, satisfactory relationship with the mother, extra-professional activities and personal psychiatric history. They also were associated with work-related factors such as the year of residency, the desired choice of specialty, the training in a general hospital with a consultation-liaison psychiatry and the perceived verbal aggression from colleagues.

Empathy dimensions’ scores, mainly those of PT and PD, were correlated with emotion regulation difficulties. In fact, PT scores were negatively correlated with five of the six emotion regulation difficulties and PD scores were positively correlated with all the six emotion regulation difficulties of the DERS. EC scores were positively correlated with PSS total score and the number of social supports. PT scores were positively correlated with SE total score and PD scores were negatively correlated with SE total score.

**Conclusions:**

Our findings underscore empathy’s complexity, revealing that in psychiatry trainees, it is shaped by contextual, emotional, and social factors. Without assessing these dimensions and mediators, empathy remains a theoretical concept rather than a teachable and improvable skill.

**Disclosure of Interest:**

None Declared

